# Detection of Usutu virus in a house martin bug *Oeciacus hirundinis* (Hemiptera: Cimicidae): implications for virus overwintering in a temperate zone

**DOI:** 10.1007/s00436-024-08325-8

**Published:** 2024-08-20

**Authors:** Silvie Šikutová, Jan Mendel, Kristína Mravcová, Romana Kejíková, Zdeněk Hubálek, Helge Kampen, Ivo Rudolf

**Affiliations:** 1 Institute of Vertebrate Biology, Czech Academy of Sciences, Kvetna 8, 603 65 Brno, Czech Republic; 2 Friedrich-Loeffler-Institut, Federal Research Institute for Animal Health, Südufer 10, 1749 Greifswald – Insel Riems, Germany; 3 Department of Experimental Biology, Faculty of Science, Masaryk University, Kamenice 753-5, 625 00 Brno, Czech Republic

**Keywords:** *Oeciacus hirundinis*, Usutu virus, House martin, Overwintering, Cimicidae, *Hirundo rustica*

## Abstract

The family *Cimicidae* comprises ectoparasites feeding exclusively on the blood of endothermic animals. Cimicid swallow bugs specifically target swallow birds (Hirundinidae) and their nestlings in infested nests. Bugs of the genus *Oeciacus* are commonly found in mud nests of swallows and martins, while they rarely visit the homes of humans. Although—unlike other cimicid species—the house martin bug *Oeciacus hirundinis* has never been reported as a vector of zoonotic pathogens, its possible role in arbovirus circulation in continental Europe is unclear. Samples of *O. hirundinis* were therefore collected from abandoned house martin (*Delichon urbicum*) nests in southern Moravia (Czech Republic) during the 2021/2022 winter season and checked for alpha-, flavi- and bunyaviruses by RT-PCR. Of a total of 96 pools consisting of three adult bugs each, one pool tested positive for Usutu virus (USUV)-RNA. Phylogenetic analysis showed that the virus strain was closely related to Italian and some Central European strains and corresponded to USUV lineage 5. The detection of USUV in *O. hirundinis* during wintertime in the absence of swallows raises the question for a possible role of this avian ectoparasite in virus overwintering in Europe.

## Introduction

The family *Cimicidae* comprises ectoparasites that feed exclusively on the blood of endothermic animals. The most important members of the family are *Cimex lectularius* and *C. hemipterus*, which infest humans and have recently resurged as a serious health problem in many parts of the world due to insecticide resistance (Štefka et al. 2022).

Swallow bugs are cimicid parasites that specifically target swallow birds and their nestlings (Hirundinidae) to feed on their blood in infested nests. The genus *Oeciacus* is commonly found in mud nests of swallows and martins, while it rarely occurs in the homes of humans. To date, three species of the genus *Oeciacus* have been described: *Oeciacus hirundinis* (Lamarck, 1816) in Eurasia, *O. montandoni* Pericart, 1972 in Romania, and *O. vicarius* Horváth, 1912 in North America (Hansel et al. 2019).

The species *O. hirundinis* is a frequent blood parasite of birds of the family *Hirundinidae*. It is commonly found in nests of swallows and martins (*Hirundo rustica*, *Hirundo daurica*, *Delichon urbicum*), but also in pigeon nests and dormouse burrows. Its reproductive cycle is adapted to its hosts, in that egg laying takes place in spring, when the birds return from their wintering grounds. The swallow bug eggs are attached to the surface of the nests, and birds carry the bugs from nest to nest as nymphs. Nestlings often die from blood loss when nests are heavily infested (Krinsky 2018).

To date, only three arboviruses associated with bird bugs have been described. Buggy Creek virus (BCRV) and the closely related Fort Morgan virus (FMV) with its variant Stone Lakes virus (STLV), two alphaviruses of the western encephalitis virus complex (family Togaviridae) which are widely distributed in western North America, are transmitted primarily by the swallow bug *O. vicarius* and amplified by avian hosts, namely the cliff swallow (*Petrochelidon pyrrhonata*) and the introduced house sparrow (*Passer domesticus*) (Pfeffer et al. 2006; Brault et al. 2009). The long-term persistence of BCRV in unfed swallow bugs (over 2 years) suggests overwintering of the virus even under unfavourable winter conditions (Brown et al. 2009, 2010). Another alphavirus isolated from *O. vicarius* is Tonate virus (TONV), a member of the Venezuelan equine encephalomyelitis virus complex occurring in northern South America (Monath et al. 1980).

Unlike other *Cimicidae* species, *O. hirundinis* has never been reported as a vector of human pathogens. However, since *O. hirundinis* attacks human hosts under certain conditions, particularly in wintertime when natural hosts are not available (Komatsu et al. 2016), it may pose a potential risk for disease agent transmission to humans. The aim of this study was therefore to screen house swallow bugs of the species *O. hirundinis* for human pathogenic arboviruses in order to check their potential role in arbovirus circulation in continental Europe.

## Materials and methods

### Cimicid collection and morphological determination

In February 2022, samples of cimicids were collected from two abandoned bird nests attached to a building in Valtice in southern Moravia (N43°36′16″, E1°26′38″). Under the roof of that building, the house martin (*Delichon urbicum*) (Passeriformes, Hirundinidae) had built typically shaped mud nests which were heavily infested with bugs. These were carefully collected with tweezers and immediately stored at − 60 °C until further processing. Morphological bug determination was performed following a classical dichotomous identification key (Usinger 1966).

### Nucleic acid extraction, amplification and sequencing

Prior to nucleic acid isolation, the bugs were surface-sterilised with 70% EtOH. A total of 96 pools (50 pools of females, 46 pools of males), consisting of three adult bugs each, were homogenised by a TissueLyser II (Qiagen, Hilden, Germany) in the presence of one stainless steel bead of 5 mm diameter (Qiagen) in 300 μl of chilled phosphate-buffered saline, pH 7.4, containing 0.4% bovine serum albumin.

For molecular identification of the bugs, genomic DNA was isolated from 100 μl of the homogenates using the QIAamp DNA Mini Kit (Qiagen) according to the manufacturer’s instructions. This was followed by PCR amplification of a 658-bp fragment of the mitochondrial cytochrome c oxidase subunit I gene (COI) using the universal barcoding primers LCO1490 and HCO2198 (Folmer et al. 1994; Hébert et al. 2003).

For virological screening, total RNA was extracted from 140 μl of bug homogenate by means of the QIAamp Viral RNA Mini Kit (Qiagen) and subjected to a continuous reverse transcriptase-polymerase chain reaction (RT-PCR) system using the OneStep RT-PCR Kit (Qiagen). All pools were tested with generic primers for alphaviruses (Eshoo et al. 2007), flaviviruses (Scaramozzino et al. 2001) and bunyaviruses (Kuno et al. 1996). Following the manufacturer’s instructions, each reaction tube contained 0.1 mM of both primers, 10 mM dNTPs, 1 μl enzyme mix, 5 μl Q-solution, 5 μl 5 × reaction buffer, 5 μl RNA extract and 7.5 μl RNase-free water to give a total reaction volume of 25 μl. PCR was performed using the Mastercycler EP Gradient 96 Well Thermal Cycler (Eppendorf, Hamburg, Germany) and the following uniform thermoprofile which turned out appropriate for all assays in previous studies (e.g. Hönig et al. 2019; Rudolf et al. 2020): reverse transcription at 50 °C for 30 min, initial denaturation at 95 °C for 15 min, followed by 40 cycles denaturation for 40 s at 94 °C, annealing for 50 s at 57 °C and extension for 60 s at 72 °C, and final extension for 7 min at 72 °C. As positive PCR controls, tick-borne encephalitis virus (strain Hypr) was used for *Flavivirus*-PCR, Ťahyňa virus (strain Ť-92) for *Bunyavirus*-PCR and Sindbis virus (strain EgAr-339) for *Alphavirus*-PCR. Positive samples were planned to be subjected to specific PCRs, but this was eventually realised only for Usutu virus (USUV) (amplification of a fragment of the NS2b-NS3 region as described by Bakonyi et al. (2004)), using strain USUV_2017_Brno as a positive control. PCR steps (pre- and post-PCR) were generally carried out in different compartments and with different equipment, so to avoid contamination. Amplified products were separated by electrophoresis in a 1.5% agarose gel stained with 1 μl/ml GelRed (Biotium, Fremont, CA, USA) and visualised under UV-light.

PCR products, both of the bug DNA amplification and the viral RNA amplification, were excised from the gel and purified by means of the ZymoClean DNA Gel Recovery Kit (Zymo Research, Irvine, CA, USA). Sanger sequencing of the purified amplicons was performed using a commercial service (SEQme, Dobříš, Chech Republic). PCR fragments were sequenced bidirectionally to ensure high-quality reads. DNA sequences were edited and aligned using the Seqman module in Lasergene v. 6.0 (DNASTAR Inc., Madison, WI, USA). The FASTA format and the BLAST search algorithm (http://www.ncbi.nlm.nih.gov/blast) from the National Center for Biotechnology Information (Bethesda, MD, USA) were used for database searches.

The single PCR-positive sample was also subjected to an attempt to isolate virus on Vero cells according to Šikutová et al. (2020).

### Phylogenetic analysis of viral RNA

For viral RNA sequence analysis, a suitable substitution model was identified based on Akaike and Bayesian information criteria using jModelTest 2.1.9 (Darriba et al. 2012). Phylogenetic analysis was then conducted using the maximum likelihood method and the general time reversible model (GTR + G + I; MEGA 7.0) (Kumar et al. 2016).

## Results and discussion

**Fig. 1:**
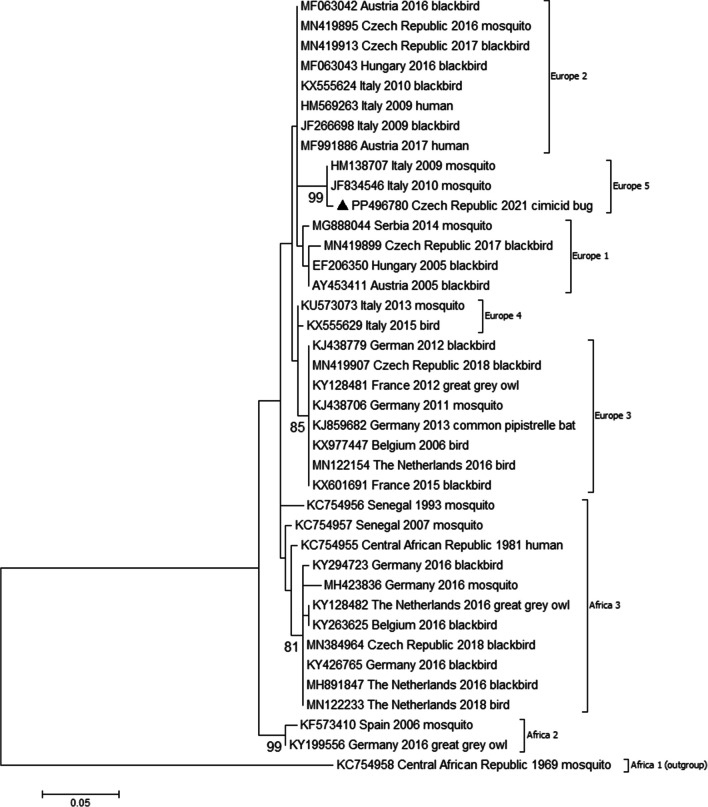
Phylogenetic maximum likelihood tree based on partial nucleotide sequences of the NS5 protein gene (264 bp) of Usutu virus. Each record consists of a particular accession number, source (human/mosquito/bird), place and year of detection/isolation. The Czech sample described here is highlighted by a black triangle. Phylogenetic analyses were conducted using the maximum likelihood algorithm with the general time reversible model (MEGA 7.0). The robustness of the tree was tested by bootstrap resampling of 1000 replicates, with values provided near the nodes (only values ≥ 80 are shown). The horizontal bars show the genetic distances

DNA sequence analysis confirmed the morphological identification of the bugs as *O. hirundinis* (COI sequence deposited in GenBank under accession number PP493925). While all 96 bug pools examined were clearly negative for alphaviruses and bunyaviruses, one pool was positive for flaviviruses. Sequence analysis of the 264-bp amplicon revealed the presence of USUV-RNA (sequence deposited in GenBank under accession number PP496780). When blasted against GenBank, this sample showed the highest sequence homology (99.6%) with USUV strains detected in *Culex pipiens* mosquitoes in Italy in 2009 (accession number HM138707) and 2010 (JF834546), respectively. Phylogenetic analysis assigned the sample to USUV lineage 5 (Fig. 1). Unfortunately, the low RNA concentration in the sample did not allow the additional amplification of other genomic virus regions or virus isolation.

USUV (Flaviviridae: *Flavivirus*) is a mosquito-borne arbovirus that circulates enzootically between birds and bird-feeding mosquitoes in Africa and emerged in Europe in 2001, causing mass mortality in blackbird populations (Weissenböck et al. 2002). In the Czech Republic, antibodies against USUV were first detected in wild birds collected from 2004 to 2006 in the framework of a serological surveillance study (Hubálek et al. 2008). In 2012, the virus could be isolated from dead blackbirds found in the city of Brno (Hubálek et al. 2014). Currently, several lineages of USUV circulate in mosquitoes and birds in different regions of the country (Hönig et al. 2019). In addition, one USUV infection was confirmed in humans during the WNV epidemic in 2018 (Zelená et al. 2021).

Naturally, cimicids are temporary ectoparasites not supposed to be carried by their hosts over long distances, although dispersal between close-by bird colonies has been demonstrated (Loye 1985; Brown and Bomberger Brown 2004). Thus, they are unlikely to represent transport vehicles for viruses between countries and continents such as ixodid ticks, for example which stay attached to their host for several days (Mancuso et al. 2022). However, the detection of USUV in avian ectoparasites may suggests a mode of virus overwintering in Central Europe in the absence of birds, including migratory species, which are considered the natural reservoirs of USUV. Overwintering of viruses in haematophagous insects occurring in temperate climate zones appears to be a common phenomenon which is regularly realised in mosquitoes, although the viruses are not necessarily found during wintertime in known or important vector species (Kampen et al. 2021; Rudolf et al. 2020; Sauer et al. 2023). In this case, USUV might persist in the cimicids over the winter and infect nestlings in spring which could then pass on the virus to biting mosquitoes to continue the cycle. This first report of USUV in cimicids therefore highlights the need for molecular screening of arthropod vectors other than mosquitoes and ticks for emerging arboviruses.

## Data Availability

Data supporting the conclusions of this article are included within the article. Representative DNA sequences have been deposited in the GenBank database under the accession numbers PP493925 (*Oeciacus hirundinis* isolate OE-1: partial cytochrome c oxidase subunit I gene) and PP496780 (Usutu virus isolate USUV-OE1/2021: partial NS5 protein gene).
